# Clinically Prepared Veterinary Students: Enhancing Veterinary Student Hands-on Experiences and Supporting Hospital Caseload Using Shelter Medicine Program

**DOI:** 10.3389/fvets.2018.00095

**Published:** 2018-05-11

**Authors:** Jacob M. Shivley, Wilson C. Brookshire, Philip A. Bushby, Kimberly A. Woodruff

**Affiliations:** Department of Clinical Sciences, Mississippi State University College of Veterinary Medicine, Mississippi, MS, United States

**Keywords:** companion animal, curriculum, educational outcomes, population medicine, shelter medicine, surgical training, veterinary medical education

## Abstract

Referral-level medicine is important in the veterinary curriculum, however veterinary students also need a solid base knowledge of clinically relevant, routine surgical and diagnostic skills to be clinically prepared after graduation. Exposure to a referral-only, or primarily referral caseload, does not always provide veterinary students with the routine hands-on experiences and competencies expected by the American Veterinary Medical Association Council on Education, the Royal College of Veterinary Surgeons, the Australian Veterinary Boards Council, or prospective employers. The aim of this descriptive study was to assess how a shelter medicine program can fill the companion animal caseload gap and create the necessary hands-on experiences considered essential in the veterinary curriculum. Pedagogical frameworks, course curriculum and design, student experiences, and student assessments were described for three core curricular areas (surgery, medical days, population medicine) of the Shelter Medicine Program at Mississippi State University. The shelter surgery experience provided a high-quality, high-volume spay/neuter environment where fourth-year students averaged 65 sterilization surgeries in two weeks and demonstrated a quantifiable decrease in surgical time. The shelter surgery experience added on average 9,000 small animal cases per year to the overall hospital caseload. Shelter medical days, where students provide veterinary care during on-site shelter visits, created opportunities for third-year students to directly interact with shelter animals by performing physical examinations and diagnostic testing, and to gain experience in developing treatment protocols and recommendations for commonly encountered problems. The shelter medical days experience averaged over 700 small animal cases per year and over 1,500 diagnostic procedures. Finally, students participated in 15 onsite shelter consultations where they obtained a working knowledge of biosecurity at a population level, including how to minimize the risk of infectious diseases spreading to healthy populations. Despite several challenges, results from this curricular program assessment support the aim that animal shelters and humane organizations offer opportunities that can be mutually beneficial for both animal organizations and veterinary students. The primary care caseload for the teaching institution was positively impacted, and students were better prepared to meet potential employers’ expectations and fulfill required core competencies in veterinary medical education.

## 1. Introduction

The American Veterinary Medical Association Council on Education (AVMA COE) standards for accreditation of veterinary schools state, in part:

“the curriculum must provide: instruction in both the theory and practice of medicine and surgery applicable to a broad range of species. The instruction must include principles and hands-on experiences in physical and laboratory diagnostic methods and interpretation (including diagnostic imaging, diagnostic pathology, and necropsy), disease prevention, biosecurity, therapeutic intervention (including surgery), and patient management and care (including intensive care, emergency medicine and isolation procedures) involving clinical diseases of individual animals and populations (1).”

Mississippi State University College of Veterinary Medicine (MSU-CVM) is located in Starkville, MS, with a human population of about 25,000. The closest metropolitan area in the state is 130 miles away (Jackson, MS with a population of about 170,000). Even with a solid referral companion animal caseload for specialty services, there can be challenges in receiving adequate numbers of primary/first opinion cases (2). While referral-level medicine is important in the veterinary curriculum, students also need a base knowledge of clinically relevant routine surgery and diagnostics (3). One method in veterinary education used to supplement the primary care caseload and provide students with greater hands-on medical and surgical experience is the utilization of shelter animals (3–6). To be prepared for veterinary practice, veterinary students need significant exposure to routine medical and surgical cases. This exposure must include hands-on opportunities to perform physical examinations, conduct and interpret routine diagnostic tests, evaluate individual and populations of animals, and perform routine surgical procedures (7, 8). The Royal College of Veterinary Surgeons (RCVS), the governing body of the veterinary profession in the United Kingdom, developed broadly-framed, essential competencies of skills that veterinary students should possess on day-one after graduation (9). The document highlights:

“A new graduate who has achieved day one competence should be capable and confident enough to practise veterinary medicine at a primary care level on their own…”

The RCVS competencies are currently under revision and are taking into account the Employability Project in Australia as well as the Association of American Veterinary Medical Colleges (AAVMC) work on Competency-Based Veterinary Education. The Australian Veterinary Boards Council (AVBC) has developed and revised its accreditation standards (10). The following is an emphasis on competence:

“competence requires more than just acquisition of technical skills: it involves applying relevant knowledge, and having the confidence and ability to transfer what has been learnt to a variety of contexts and new unpredictable situations.”

Exposure to a referral only, or primarily referral caseload, does not provide veterinary students with the routine hands-on experiences that are implied in the AVMA COE standards for accreditation, expected by RCVS day-one competencies, required by the AVBC for students to have confidence and ability to transfer technical skills, and desired by prospective employers (1, 2, 8, 9, 11).

The MSU-CVM Shelter Medicine Program was established with a goal of filling the companion animal caseload gap and creating hands-on opportunities with shelter animals (dogs and cats) to perform high-quality high-volume spay/neuter (HQHVSN), physical examinations, and basic diagnostic tests. These opportunities are aimed at developing core competencies and technical skills as outlined by organizations across the world.

The Shelter Medicine Program is delivered in three core areas: shelter surgery, shelter medical days, and population medicine. Within these core areas, there are three pedagogical frameworks that provide the foundations for these learning experiences. Experiential learning, as first described by Dewey (12) and later developed into a modern theory by Kolb (13), is defined as learning through reflection on doing. Students are provided with concrete experiences, followed by self-reflection with direct feedback from faculty and are then given many opportunities for practice and improvement. This naturally leads into the second theoretical framework heavily used in the Shelter Medicine Program, deliberate practice as described by Ericsson (14, 15). This framework describes engaging in deliberate practice with feedback during training. Although experts aren’t developed in the short time students are with the Shelter Medicine Program, the tenets of the deliberate practice framework are provided. Finally, all services of the Shelter Medicine Program directly engage communities across Mississippi while providing best practice, high quality clinical instruction to veterinary students. The “integration of the accomplishment of a public task with conscious educational growth” fulfills the principles of the service-learning framework as outlined by Sigmon (16).

The aim of this article is to describe how a comprehensive shelter medicine program incorporated into the veterinary curriculum provides students with the hands-on experiences considered essential in veterinary education.

## 2. Core Area Curriculum and Course Design

### 2.1. Shelter Surgery

The MSU-CVM Shelter Medicine Program has two mobile veterinary units that provide HQHVSN services to 26 different shelters and rescue groups in central and north Mississippi. This program provides two opportunities for students to increase surgical skills and confidence (Table 1).

**Table 1:** Summary of Shelter Experiences and Learning Objectives.

**Curricular Year**	**Program**	**Duration**	**Students involved**	**Major learning objectives**
3rd year	Shelter Spay/Neuter	2 days	All third-year students	Hands-on surgical experience.Understand issues shelters face.Understand pet over-population.Understand disease issues in shelter environment.
3rd year	Population Medicine rotation	3 weeks	All third-year students	Learn how population level protocols and procedures affect animal health outcomes.Learn to think critically about causation when investigating animal health outcomes.Become familiar with diagnostic tests in population-based disease investigations.Understand how to use data (evidence) to investigate and monitor population health.Learn strategies for disease control and prevention.Become proficient in evidence-based approaches to solving complex animal health problems.
3rd year	Shelter Medical Days	3 days	All third-year students	Become proficient in canine and feline physical examination techniques.Learn how to perform point of care diagnostic procedures.Develop treatment plans for individual shelter animals.Understand population medicine concepts as they relate to individual animal care.Recognize the severity of pet overpopulation and how veterinarians can play a role in solving the problem.Understand the standards of care in animal shelters.
4th year	Shelter Spay/Neuter	2 weeks	Elective experience 80% of fourth-year students enroll	Become proficient and efficient in spay/neuter techniques.Recognize the severity of pet overpopulation and how veterinarians can plan a role in solving the problem.Understand the standards of care in animal shelters.Understand the veterinary medical care guidelines for spay/neuter programs.

#### 2.1.1. Third-Year Student Experiences

As part of a 6 week Community Veterinary Services (CVS) rotation, third-year students are required to spend 2 days on the mobile units learning HQHVSN techniques under the direct supervision of a shelter medicine faculty member or resident. Learning objectives for third-year students include:

- Perform multiple sterilization surgeries with direct surgical guidance, verbal and written feedback.
- Become familiarized with the issues that animal shelters face.
- Exposure to the problems encountered in animals confined to animal shelters.
- Develop an increased understanding of the issues surrounding overpopulation of unwanted pets.

Prior to their first surgery day, all CVS students are required to view instructional videos of HQHVSN techniques in both pediatric and adult dogs and cats. The surgical techniques used are consistent with the standards outlined by the Association of Shelter Veterinarians (ASV) 2016 (17). Students then perform surgeries with a faculty surgeon scrubbed in as their assistant, guiding them through the procedures and intervening to prevent complications. Furthermore, they are exposed to current research projects evaluating surgical techniques in HQHVSN. Students are  assessed on multiple parameters and are provided with direct feedback both during surgery and after each trip. Preparation for surgery and student surgical skills are evaluated using an objective structured assessment (Supplementary Table S1).

#### 2.1.2. Fourth-Year Student Experiences

Building upon the experience gained in the CVS rotation, the Shelter Medicine spay/neuter elective is a 2 week course in shelter surgery providing more in-depth training. Fourth-year students are given the time needed to become proficient and efficient in HQHVSN techniques under direct supervision and guidance of shelter medicine faculty. The course has two main goals: teaching and community service. Learning objectives include:

- Become proficient and efficient in spay/neuter techniques.
- Recognize the severity of pet overpopulation and how veterinarians can plan a role in solving the problem.
- Understand the standards of care in animal shelters.
- Understand the veterinary medical care guidelines for spay/neuter programs.

The elective is available nearly year-round and can enroll 115 students a year. A typical week consists of 4 trips on the mobile veterinary units to local shelters and 1 day of individual study. Working with faculty and house officers, students participate in the shelter spay/neuter programs at various animal shelters, and in community spay day programs when available. Independent of the onsite shelter activities, the students:

- View spay/neuter instructional videos prior to the first trip.
- Familiarize themselves with the drug protocols used on the mobile units.
- Read the document The Association of Shelter Veterinarians Veterinary Medical Care Guidelines for Spay-Neuter Programs (17).
- Read the document Guidelines for Standards of Care in Animal Shelters (18).
- At the midpoint of the course, submit a self-evaluation to the instructor.
- At the end of the course, submit an examination to the instructor.

On the first day of the rotation, students scrub in with a faculty member and are guided through an adult dog neuter, adult dog spay, puppy spay, and cat spay. Pediatric spay/neuter (aged between 6 and 16 weeks) is taught and encouraged in the animal shelter setting (17). During this time, student preparation is directly assessed by the faculty member through questioning of surgical anatomy and procedural details. Afterwards, most students perform surgeries unassisted, while still under direct supervision of a faculty member. Student-performed surgeries adheres to the Mississippi Veterinary Practice of 2008 (USA), the AVMA Model Veterinary Practice Act of 2017, and Veterinary Surgeons Act of 1966 (UK). If complications arise, students are guided through appropriate measures to safely resolve the issue. Faculty members will step in to complete the surgery if the patients’ health is at risk or if the surgery lasts longer than an hour. Students are exposed to anesthesia, anesthetic monitoring, and pain management, but these are not the primary learning objectives for the fourth-year shelter experience as these are thoroughly taught in a third-year required anesthesia rotation.

Post-operative care for surgical patients must be accomplished by shelter personnel due to the mobile nature of the program. To ensure high-quality medical care for all patients, each shelter is required to have a local veterinarian agree to manage post-operative medical needs. Follow-up from MSU-CVM faculty is accomplished through routine email and phone communications to shelter personnel and local veterinarians when complications do occur.

Student evaluations are accomplished through direct observation and a final examination. A mid-point self-evaluation encourages students to reflect on their strengths and weaknesses during surgery, and to identify areas where improvement is needed. A faculty member reviews each evaluation and responds with feedback on ways to improve during the second week. Final grades for the two-week rotation are determined by 80% instructor evaluation and 20% final examination. All involved faculty members provide an evaluation based on an objective structured assessment (Supplementary Table S2). The examination covers topics from the ASV guidelines for standards of care in animal shelters and the standards of care for spay/neuter programs.

#### 2.1.3. Data Recorded

The number and type of surgical cases are recorded for each student. Medical records of all surgical cases are entered into a computerized system and are counted as part of the overall teaching hospital caseload.

### 2.2. Shelter Medical Days

Shelter medical days were created to provide students with practical diagnostic, technical, and examination skills. Third-year students attend 2–3 shelter medical days during their required 6 week CVS rotation (Table 1). During a shelter medical day trip, 3–5 veterinary students receive hands-on training though onsite shelter visits with a faculty member. Students interact directly with the supervising veterinarian during the examination, diagnostic testing, and development of treatment protocols and recommendations. In addition to direct patient care, students gain experience and skills in biosecurity, infection control within a population of animals, and animal behavior. Learning objectives include:

- Become proficient in canine and feline physical examination techniques.
- Learn how to perform point of care diagnostic procedures.
- Develop treatment plans for individual shelter animals.
- Understand population medicine concepts as they relate to individual animal care.
- Recognize the severity of pet overpopulation and how veterinarians can play a role in solving the problem.
- Understand the standards of care in animal shelters.

#### 2.2.1. Student Hands-on Experiences

Students visit numerous types of shelter models including privately and municipally funded shelters and both limited admission and open admission facilities. Shelter medical days begin with a brief introduction to the shelter system by the faculty shelter veterinarian and shelter staff. Students perform a brief walk-through evaluation of animals housed in the facility.

Animals are selected for evaluation by students based on the needs of the shelter. Students perform intake examinations, routine wellness examinations, and sick-animal examinations under the direct supervision of a veterinarian. Students have access to the equipment needed for physical examinations and common point-of-care diagnostics. Physical examination equipment includes personal protective equipment, ophthalmoscopes, otoscopes, video otoscopes, thermometers, and stethoscopes. Point-of-care diagnostic supplies include needles, syringes, slides, microscopes, fecal flotation solution, scalpel blades, clear plastic tape, cytology staining liquids, blood collection tubes, and a Wood’s lamp. Additional point-of-care diagnostics including parvovirus ELISA tests, heartworm antigen ELISA tests, and FIV/FELV ELISA tests are provided by the shelters. With these supplies, students perform and become proficient in numerous point-of-care diagnostics including fine needle aspiration with cytology, ear swab cytology, impression smear cytology, skin scrapings, tape-prep cytology, venipuncture, Wood’s lamp evaluation, fecal oocyte identification, and wet-mount microfilaria evaluation. Students also perform routine procedures such as vaccine administration, anthelmintic administration, ear cleaning, and wound care. Under the supervision of a veterinarian, students use the information obtained from animal histories, physical examinations, and diagnostic tests to make further diagnostic and treatment recommendations for the animals in the care of the shelter. Students are given direct feedback through hands-on instruction during shelter visits along with formal evaluations using an objective structured assessment (Supplementary Table S3). Students receive 40 total possible points for shelter medical days that account for 10% of their overall CVS rotation grade.

#### 2.2.2. Biosecurity

In addition to direct patient care, students also gain experience and knowledge of biosecurity during shelter medical days. Biosecurity strategies are critically important for animal shelters and include policies and procedures to prevent and control disease. Commonly discussed biosecurity topics include sanitation procedures, disinfection procedures, visitor policies, workflow policies, and facility design. Faculty lead students through the shelter for a biosecurity walk-through evaluation. After discussing biosecurity as a group, students participate in a biosecurity photo-scavenger-hunt where good and poor illustrations are documented in photographic format. For example, incorrect use of disinfection products is a common example of poor biosecurity in animal shelters. Students lead problem-based discussions in a round-table format to solve biosecurity issues that they encountered during the photo-scavenger-hunt.

#### 2.2.3. Behavioral Health

At least 1 trip for each student is a shelter behavioral health day. Students are exposed to routine behavioral health, safe/low stress handling techniques, and enrichment techniques. Shelter behavioral health days operate similarly to shelter medical days, but >50% of the day is spent working through behavioral concepts and cases. Students are accompanied to the shelter by a MSU clinical instructor in behavioral medicine. Students are introduced to reading animal body language, non-stressful handling techniques, enrichment strategies, behavior assessments, and basic training strategies. Students also work through more complex behavior cases and develop treatment protocols involving training, enrichment, and sometimes pharmacologic intervention.

#### 2.2.4. Data Recorded

**Figure 1:**
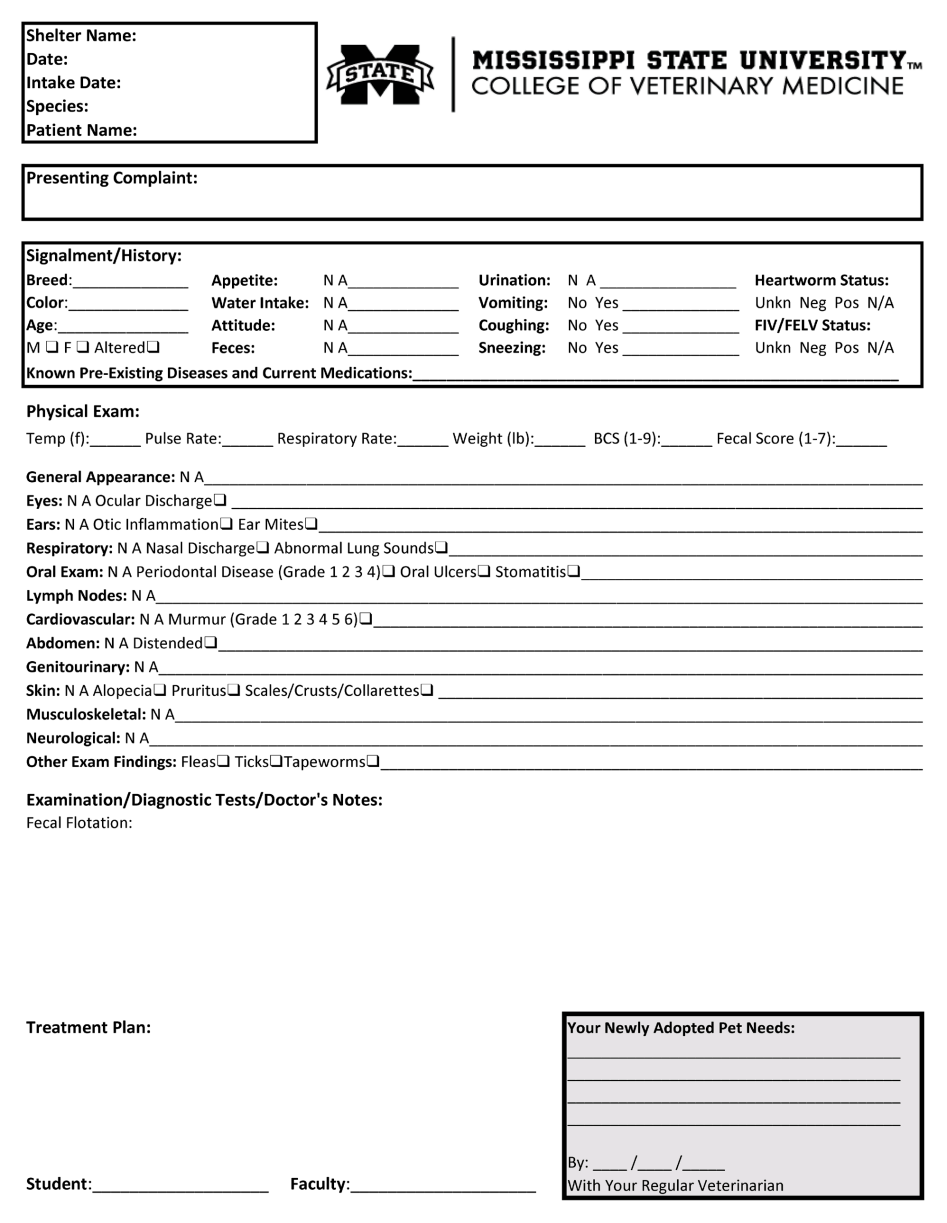
Physical Examination Form. The physical examination form used on shelter medical days.

Medical notes created by the students are recorded on a physical examination form (Figure 1) and inserted into the patient’s file at the shelter for future use by the shelter, potential adopters, and veterinarians. Physical examination findings, diagnostic procedure results, and treatment recommendations are also cataloged into a digital format for data collection and case number reporting. The number and types of cases are recorded for each student. All medical cases are counted as part of the overall teaching hospital caseload.

### 2.3. Population Medicine

Veterinarians, no matter what their area of interest or expertise, will find themselves addressing animal health issues both at the individual and population level (Table 1). Private practitioners whose main responsibilities lie with treating and preventing disease in individual patients need a working knowledge of biosecurity on a population level in order to minimize the risk of infectious diseases spreading to healthy populations. Students receive this training in a required 3 week Population Medicine rotation and learning is guided by six core learning objectives:

- Learn how population level protocols and procedures affect animal health outcomes.
- Learn to think critically about causation when investigating animal health outcomes.
- Become familiar with diagnostic tests in population-based disease investigations.
- Understand how to use data (evidence) to investigate and monitor population health.
- Learn strategies for disease control and prevention.
- Become proficient in evidence-based approaches to solving complex animal health problems.

Students are evaluated at the beginning of the rotation with a pretest to determine their understanding of the six core learning objectives. This rotation teaches the concepts across a variety of species, including cattle, deer, zoo animals, swine, catfish, chickens, dogs, and cats. The shelter program plays a large role in this rotation, as the students are on-site in a shelter for at least 3–4 trips per rotation. The students’ first shelter visit of the rotation is focused on providing care for dogs at intake through preventive care, screening for signs of disease, and establishing or following biosecurity protocols. The second visit to another shelter exposes them to a variety of shelter systems. The culmination of their time in shelters during the rotation occurs when the students take part in on-site targeted consults, comprehensive shelter consults, or outbreak investigations.

#### 2.3.1. Shelter Consultation Experience

Shelters reach out to the faculty of the Population Medicine rotation seeking answers to questions regarding disease control, shelter management, or animal health needs. Students play an important role in the consultation or outbreak investigation. Guided by faculty members, they collect data and gather specific information regarding the concerns of the shelter employees or the disease process of interest. Following participation in the consultation or outbreak investigation, the students analyze the data, gather credible scientific literature, and form recommendations regarding the issues. It is the responsibility of the students to draft an initial recommendation letter that, after review and editing by faculty, will be submitted to the shelter.

#### 2.3.2. Data Recorded

Records are kept of all consultations. Written reports are generated by the students, edited by faculty, and forwarded to the respective shelter(s).

## 3. Results

### 3.1. Shelter Surgery

The mobile unit program has sterilized over 70,000 animals since its inception in 2007 effectively adding on average 9,000 cases a year to the overall hospital caseload. The base small animal hospital case load averages 10,000 cases per year. While providing community and statewide service is a great benefit of the program, the primary value is increased surgical skills and confidence of the veterinary students.

Students perform the clear majority of the spay/neuter surgeries on the mobile units. Each third-year CVS student averages 15–20 sterilization surgeries in two trips. On average, each fourth-year student performs 65 sterilizations during their two-week rotation (Table 2). With this quantity of surgical procedures students experience an increase in surgical confidence, efficiency, and proficiency. A retrospective study performed by the authors and their team examined 1,132 dog spays completed by 86 students. Surgical times ranged from 8 to 153 min (mean 43.9). Linear regression using a generalized linear mixed model demonstrated that the average surgical time for a student’s first solo dog spay (intercept) was 50.6 min and that surgical times decreased 0.61 min (*p* value < .0001) for each consecutive dog spay performed by the individual student (Table 3).

**Table 2:** Example Case Log for Fourth-year Student on Shelter Surgery Elective.

Date	Shelter	Kitten neuter	Cat neuter	Kitten spay	Cat spay	Puppy neuter	Dog neuter	Puppy spay	Dog spay	Total
8/22/16	West Point				6	3		2	1	12
8/23/16	Macon		2		1	1		2	2	8
8/25/16	HB		3		2		1	1		7
8/26/16	Aberdeen		1		5		2	1	2	11
8/30/16	Starkville		2		4	2	1	1	1	11
9/1/16	Indianola		3		5		2	1	1	12
9/2/16	Indianola				1	6		3		10
										71

**Table 3:** Linear Regression Using a Generalized Linear Mixed Model. Analysis for Student Surgical Time.

Effect	Estimate	SE	DF	t Value	PR > |t|
Intercept	50.5838	1.2682	85	39.89	<.0001
order	−0.6070	0.08192	1044	−7.41	<.0001

Since the acquisition of a 2nd mobile unit in 2012, 80% of all third-year students enroll in the 2 week Shelter Medicine spay/neuter elective during their fourth year. While the Shelter Medicine Program shelter program provides valuable services to shelters and humane groups throughout central and north Mississippi, the true value is in providing hands-on experiences and deliberate practice for students to improve surgical skills and knowledge. Allowing students to perform the surgeries and procedures without direct faculty assistance instills confidence and allows them to take responsibility for the surgical outcomes. Quantitative data via instructor evaluation using objective structured assessments are provided in Supplementary Table S4. Qualitative data is collected after each course via student evaluations using an online evaluation portal. Fourth-year students are overwhelmingly positive about the surgical experiences gained:

“Wonderful rotation that allowed me to gain confidence and experience in spays and neuters. I do not know what the first months out in practice would be like without having this valuable experience under my belt. Very thankful to all the clinicians and technicians that make this rotation one of the most valuable (in my opinion) rotation in vet school.”

### 3.2. Shelter Medical Days

Shelter medical days provide a large number of cases for veterinary students at MSU-CVM. Each student in the MSU-CVM class of 2016 evaluated an average of 8 patients and performed an average of 16 procedures/diagnostic tests during their CVS medical/behavioral days. As a whole, the class of 2016, consisting of 83 students, evaluated 674 patients and performed 1,359 procedures/diagnostic tests. Each student in the class of 2017 evaluated an average of 10 patients and performed 21 procedures/diagnostic tests. The class of 2017, consisting of 81 students, evaluated 785 patients and performed 1,691 procedures/diagnostic tests.

In the Shelter Medical Days experience, while many animals are young and relatively free of serious disease processes, the opportunity to gain a solid knowledge base of normal physical examination findings builds a foundation for future cases. Furthermore, the repetition of multiple cases aids in improving diagnostic and technical skills. Students that participate in the shelter medical days are very positive about the experience and leave with greater confidence in performing physical examinations, point-of-care diagnostics, and treatment plan development. The following is an excerpt from a student evaluation:

“This has been my absolute favorite thing to do in vet school. We get hands on experience and I felt like I was actually making an impact and using the skills I have learned… I felt that all the clinicians were encouraging and helped answer questions you had, while also trying to get you to think and problem solve on your own. I wish I could do this every day!”

### 3.3. Population Medicine

Between June 2016 and August 2017, 15 onsite consultations were conducted with shelters or animal holding facilities. These onsite sessions consist of comprehensive, follow-up, or targeted consultations. Comprehensive consultations typically involve assessing many areas of shelter operations and providing recommendations for improvement. Follow-up consultations occur 1–6 months following an initial comprehensive consultation to provide additional assessment and feedback. Targeted consultations are often for a specific disease outbreak or area of shelter operations such as disinfection protocols.

Between June 2016 and August 2017, 115 students took part in the rotation. Students are evaluated with subjective faculty evaluations as well as an end of rotation final exam. Subjective evaluations assess professionalism, record keeping, communication, biosecurity knowledge, knowledge of diagnostic tests, and ability to critically evaluate scientific literature. The final exam is multiple choice and is designed to evaluate the students’ understanding of the rotation’s core learning objectives. Finally, the students learn another important principle: concise and constructive communication.

## 4. Discussion

The aim of this article was to describe a comprehensive shelter program incorporated into the veterinary curriculum and how it provides students with the hands-on experiences considered essential in veterinary education as prescribed by veterinary governing bodies globally. The results support this aim and shows the quantity of student experience and how a shelter program can add considerable numbers to a small animal hospital case load.

The services of the MSU-CVM Shelter Medicine Program are offered to shelters free of charge. The College of Veterinary Medicine funds 3.4 faculty positions committed to the shelter program. Additional faculty from the Population Medicine Service and Community Veterinary Services participate on an intermittent basis in providing epidemiology, population medicine, and behavioral expertise. Funding for three full-time veterinary technicians and four student workers, vehicle maintenance, and all medical and surgical supplies are funded through grants and donations.

Challenges exist in the MSU-CVM Shelter Medicine Program. Providing mobile veterinary care to area shelters across central and north Mississippi requires a flexible approach by teaching faculty and staff. Three full-time faculty along with available house officers are responsible for approximately 9,000 teaching cases per year. This requires creative scheduling to ensure quality teaching and contact time for the students involved. Under the umbrella of the Shelter Medicine Program, there are many activities — often each happening at the same time, but in different locations. Therefore, having the necessary veterinary and technical man-power is required to ensure program success. Objective structured assessments are a priority for the faculty, but every student cannot be guaranteed the same learning experiences due to the variable caseload between animal shelters. For example, one student may perform three adult dog castrations during their two-week surgical elective while another student may perform 20 adult dog castrations. The current rubrics used for the objective structured assessments have been valuable, but some modifications are being considered. The first is to change the pass/fail threshold criteria. Currently the pass/fail options are based on professionalism and behavior; ideally these would be shifted to skill-based criteria founded on the competencies needed to sterilize an adult female dog. Taking this idea even further, in order to build a competency-based veterinary curriculum and assessment, a new framework is on the horizon for veterinary medicine. Entrustable Professional Activities (EPAs) will likely become required for veterinary teaching institutions in the near future, therefore effort is being made now to transition into teaching and assessing students on shelter medicine rotations using this framework.

Beyond personnel, vehicle and trailer maintenance and mechanical failures can become difficulties, sometimes completely disrupting a day’s activities. Routine preventive maintenance must be performed, therefore a 2 week hiatus from road trips in July and 4 weeks in December provides the necessary time for servicing needs.

Relying on shelters and humane organizations to provide animals and cases for student experience can be a frustrating scenario. Shelter intake may be low during a certain month and students may receive less surgical opportunities. Similarly, students can become frustrated because they do not get to see the outcome of surgical and medical cases. Valuable information can be obtained through managing post-operative complications, but relying on a mobile surgical model can withdraw students from these learning situations. Local veterinarians do manage these complications, but this often times removes students from the process. Furthermore, one group of students may handle an initial population medicine consult by delivering the initial communication and recommendations, while a later group provides the follow up visit and benefits from seeing the end results.

The faculty of the Shelter Medicine Program continuously strive to improve the student experience while assisting the shelters in their community. A building on the MSU-CVM campus is currently being renovated into a fully operational HQHVSN clinic. This will serve as an alternative to one of the mobile veterinary clinics. Animal shelters will transport animals to the facility to receive sterilization services. This model helps to relieve some of the maintenance and scheduling issues encountered with the mobile veterinary clinic. It also provides an opportunity to work with shelters and humane organizations that are beyond the travel radius of the mobile veterinary clinic. Additionally, there are plans to expand the opportunities for shelter medicine consultation and outbreak investigations through a fully equipped shelter diagnostic and teaching lab. This lab will include the necessary supplies and equipment for complete physical examinations, fecal centrifugation, skin/ear cytology, urinalyses including sediment evaluation, infectious disease testing, and digital medical records. Finally, a shelter medicine focused continuing education meeting is planned for shelter personnel, veterinarians, and veterinary technicians in the region and will be delivered by the MSU-CVM Shelter Medicine Program.

## 5. Conclusion

Animal shelters and humane organizations offer opportunities that can be mutually beneficial for both the animal organization and veterinary students. Students gain valuable experience that prepares them for their career while satisfying many core competencies for veterinary students outlined by organizations worldwide, and shelters are provided with animal health care and consultative services that otherwise would not have been attainable. The overall caseload for the teaching institution is positively impacted and students are better prepared to meet potential employer’s expectations.

## Author Contributions

PB began the Shelter Medicine Program. JS conceived the idea of this article. JS, WB, PB, and KW maintain and organize records and data sets. JS, WB, PB, and KW wrote sections of the manuscript. KW performed the statistical analysis. All authors approved submission.

## Conflicts of Interest

The authors declare that the research was conducted in the absence of any commercial or financial relationships that could be construed as a potential conflict of interest.
