# Supplementary material for: Clinically Prepared Veterinary Students: Enhancing Veterinary Student Hands-on Experiences and Supporting Hospital Caseload Using Shelter Medicine Program
Source: Front Vet Sci. 2018 May 11;5:95. doi: 10.3389/fvets.2018.00095 (PMC5958676; doi:10.3389/fvets.2018.00095)
Supplement: Supplementary file 1 [file Table1.DOCX]

Supplementary Material

**Clinically Prepared Students Using Shelter Medicine**

**Jacob M Shivley ^1^*, Wilson C Brookshire^1^, Philip A Bushby^1^ and Kimberly A Woodruff^1^**

^1^Department of Clinical Sciences, Mississippi State University College of Veterinary Medicine, Mississippi State, MS, USA

***Correspondence**: j.shivley@msstate.edu

| **Skill** | **1** | **2** | **3** | **4** | **5** |
| --- | --- | --- | --- | --- | --- |
| Understanding anatomy | Not familiar with surgical anatomy | Struggles with understanding of surgical anatomy | Understands the anatomy of most structures encountered | Understands all major structures encountered | Full understanding of anatomy related to surgery |
| Understanding surgical procedure | Not familiar with the surgical procedure | Struggles with understanding of surgical procedure | Understands the basic surgical procedure | Understands the surgical procedure completely | Full understanding, including alternatives and potential complications |
| Attention to asepsis | Does not understand/ follow aseptic technique | Problems with aseptic technique; required repeated instructor intervention | Pays attention to aseptic technique; minor breaks corrected by instructor | Attention to asepsis; breaks in asepsis noted and corrected | Absolute attention to asepsis; no breaks in sterile technique noted |
| Prevention/control of hemorrhage | Does not follow appropriate techniques for prevention/control of hemorrhage | Problems with management of hemorrhage; required repeated instructor intervention | Minor problems with hemorrhage that required intervention by instructor | Consistently manages hemorrhage with no risk to the patient | Consistently prevents hemorrhage before it occurs |
| Minimizing tissue trauma | Tissue severely damaged unnecessarily | Inadvertent trauma to structures not routinely involved in the surgical procedure | Frequent unnecessary tissue manipulation resulting in considerable tissue trauma | Occasional tissue manipulation; slight increase in tissue trauma | Minimal tissue trauma |
| Efficiency of surgical skills | Considerable wasted motion, indecisive; surgical time excessive | Frequent wasted motions/indecision | Occasional wasted motions/indecision | Surgery performed efficiently; minimal wasted motion | Surgery performed with utmost efficiency; no wasted motions |
| Surgical competence/confidence | Confidence in surgical skills is unwarranted; lacks basic surgical skills necessary for minor/routine procedures | Lacks confidence in surgical skills; lacks basic surgical skills necessary for minor/routine procedures | Competent in many aspects of surgical procedure | Competent in most aspect of surgical procedure; minor problems as described below | Confident in surgical skills; very competent skills |
|  |  |  |  |  |  |
|  | Pass | | Fail | |  |
| Patient care | All students are expected to provide thorough and competent care for their patients. This is a threshold skill. | | | | |
| Professional attributes | All students are expected to communicate effectively with clients, technicians, and faculty, to complete medical records and surgery reports accurately and expeditiously and to act professionally at all times. | | | | |

**Supplementary Table 1**. Objective Structured Assessment of Third-Year Students. Each skill is graded 1-5 with a total of 35 points possible.
