# Supplementary material for: Clinically Prepared Veterinary Students: Enhancing Veterinary Student Hands-on Experiences and Supporting Hospital Caseload Using Shelter Medicine Program
Source: Front Vet Sci. 2018 May 11;5:95. doi: 10.3389/fvets.2018.00095 (PMC5958676; doi:10.3389/fvets.2018.00095)
Supplement: Supplementary file 2 [file Table2.DOCX]

Supplementary Material

**Clinically Prepared Students Using Shelter Medicine**

**Jacob M Shivley ^1^*, Wilson C Brookshire^1^, Philip A Bushby^1^ and Kimberly A Woodruff^1^**

^1^Department of Clinical Sciences, Mississippi State University College of Veterinary Medicine, Mississippi State, MS, USA

***Correspondence**: j.shivley@msstate.edu

| Student |  |  | | |
| --- | --- | --- | --- | --- |
| Date |  |  |  |  |
| Grade |  |  |  |  |
|  |  |  |  |  |
| **Surgical Skills** | **Description** | **Value** | **Score** | **Comment** |
| Understands each procedure | Understands the basics of each procedure at the start of the rotation (i.e. has thoroughly reviewed the PowerPoints and videos). | 10 |  | Remember, every skill you need for virtually any soft-tissue surgery commonly done in private practice, you use in a spay. So if you are comfortable doing spays, you have the skills to perform any routine soft-tissue surgery! |
| Applies what is learned | Applies what is learned each day to subsequent surgeries, i.e. doesn’t repeat mistakes. | 10 |  |  |
| Detail | Thinks about what he/she is doing. Pays attention to detail. Performs tasks appropriately. | 10 |  |  |
| Efficient surgical skills | Adopts efficient techniques. Shows improvement in efficiency over the course. | 10 |  |  |
| Maintains hemostasis | Effectively prevents hemorrhage. | 10 |  |  |
| Minimizes tissue trauma | Treats tissues gently. | 10 |  |  |
| Maintains asepsis | Pays attention to maintaining asepsis. | 10 |  |  |
| Closures | Secure body wall closures. Consistently closes dead space. Good skin to skin apposition with subcuticular patterns. | 10 |  |  |
| Total Points from Rotation | | 80 |  |  |
| Exam | | 20 |  |  |
| Total points | | 100 |  |  |
|  |  |  |  |  |
| Professional Values/ Behavior* |  | pass | fail |  |
| Animal care | Treats all animals with respect. Handles animals appropriately. | √ |  |  |
| Attendance punctuality | Arrives on time. | √ |  |  |
| Overall initiative | Pitches in to get all the work done. | √ |  |  |
| Attitude | Positive friendly attitude. | √ |  |  |
| Professionalism | Acts professional. Positive representative of Mississippi State and the Veterinary Profession. | √ |  |  |

**Supplementary Table 2.** Objective Structured Assessment of Fourth-year Students.
