# Supplementary material for: Clinically Prepared Veterinary Students: Enhancing Veterinary Student Hands-on Experiences and Supporting Hospital Caseload Using Shelter Medicine Program
Source: Front Vet Sci. 2018 May 11;5:95. doi: 10.3389/fvets.2018.00095 (PMC5958676; doi:10.3389/fvets.2018.00095)
Supplement: Supplementary file 3 [file Table3.DOCX]

Supplementary Material

**Clinically Prepared Students Using Shelter Medicine**

**Jacob M Shivley ^1^*, Wilson C Brookshire^1^, Philip A Bushby^1^ and Kimberly A Woodruff^1^**

^1^Department of Clinical Sciences, Mississippi State University College of Veterinary Medicine, Mississippi State, MS, USA

***Correspondence**: j.shivley@msstate.edu

| **Skill** | **1** | **2** | **3** | **4** | **5** |
| --- | --- | --- | --- | --- | --- |
| Understanding ASV Shelter Guidelines | Not familiar at all with ASV Shelter guidelines. | Struggles with understanding of guidelines. | Understands most of the basic shelter principles in the guidelines. | Understands all major principles encountered in the ASV guidelines. | Full understanding of guidelines; able to apply principles to current shelter. |
| Understanding biosecurity principles/photo scavenger hunt | Not familiar with biosecurity principles. | Struggles with understanding of biosecurity principles. | Understands basic biosecurity principles. Limited understanding of fine details biosecurity principles. | Understands biosecurity principles completely, familiar with fine details. | Full understanding of all aspects of shelter biosecurity principles and application to current shelter. |
| Physical examination | Does not know how to perform a physical examination. | Attempts a PE, but struggles, requiring repeated instructor intervention for basic principles. | Performs PE well, but occasionally misses body systems or abnormal findings. | Performs PE well. | Performs PE extremely well, covering all body systems and abnormal findings. |
| Fecal float technique and analysis | Does not understand how to perform fecal float or analysis. | Problems with fecal float technique and analysis. Required repeated instructor intervention. | Minor problems with fecal float technique and analysis. Misses most organisms with analysis. | Fecal float and analysis performed well. Most organisms ID'd correctly. | Performs fecal float and analysis extremely well. All organisms ID'd correctly. |
| Differential diagnosis | No effort to create differential diagnosis when warranted. | Differential diagnosis attempted. | Differential diagnosis created, but incomplete. Major diseases not included. | Differential diagnosis list created, only minor diseases not included. | Excellent differential diagnosis list; complete and thorough. |
| Diagnostic plan/abilities | Does not understand how to formulate diagnostic plan. | Problems with forming plan and unable to perform recommended tests. Required repeated instructor intervention. | Minor problems with forming plan and completing diagnostics tests. Only some organisms identified correctly. | Consistently forms excellent plan, but occasionally has problems with performing diagnostic tests. Most organisms ID'd correctly. | Creates diagnostic plan and performs diagnostics very well. All organisms ID'd correctly. |
| Treatment plan | No effort to create treatment plan when warranted. | Treatment plan attempted, but instructor intervention necessary. | Treatment plan created, but incomplete and major diseases not addressed; instructor intervention necessary. | Treatment plan created, minor details addressed and corrected. | Excellent treatment plan; complete and thorough. Able to present plan to shelter personnel. |
| Preventative medicine / general wellness | No effort to create general wellness plan. | Lacks understanding of general wellness (vacc. protocols, etc.) | Competent in many aspects of preventive medicine and wellness care. | Competent in most aspects of preventive medicine and wellness. | Confident and competent in creating and recommending general wellness plans to shelter personnel. |
|  |  |  |  |  |  |
| Comments |  | | | | |

**Supplementary Table 3**. Objective Structured Assessment for CVS Medical Days. Each skill is graded 1-5 with a total of 40 points possible.
