# Supplementary material for: Clinically Prepared Veterinary Students: Enhancing Veterinary Student Hands-on Experiences and Supporting Hospital Caseload Using Shelter Medicine Program
Source: Front Vet Sci. 2018 May 11;5:95. doi: 10.3389/fvets.2018.00095 (PMC5958676; doi:10.3389/fvets.2018.00095)
Supplement: Supplementary file 4 [file Table4.DOCX]

Supplementary Material

**Clinically Prepared Students Using Shelter Medicine**

**Jacob M Shivley ^1^*, Wilson C Brookshire^1^, Philip A Bushby^1^ and Kimberly A Woodruff^1^**

^1^Department of Clinical Sciences, Mississippi State University College of Veterinary Medicine, Mississippi State, MS, USA

***Correspondence**: [j.shivley@msstate.edu](mailto:j.shivley@msstate.edu)

| **Surgical Skills** | **Academic Year 2015-16** | | | | |
| --- | --- | --- | --- | --- | --- |
| **Question** | **Scale** | **Total Answered** | **Total Unanswered** | **Mean** | **SD** |
| Understands the basics of each procedure at the start of the rotation (i.e. has thoroughly reviewed the PowerPoints and videos). | 10 | 159 | 10 | 9.82 | 0.46 |
| Applies what is learned each day to subsequent surgeries, i.e. doesn't repeat mistakes. | 10 | 167 | 2 | 9.43 | 0.89 |
| Thinks about what he/she is doing. Pays attention to detail. Performs task appropriately. | 10 | 168 | 1 | 9.43 | 0.79 |
| Adopts efficient techniques. Shows improvement in efficiency over the course. | 10 | 167 | 2 | 9.16 | 0.93 |
| Effectively prevents hemorrhage. | 10 | 168 | 1 | 9.50 | 0.66 |
| Treats tissues gently. | 10 | 168 | 1 | 9.55 | 0.72 |
| Pays attention to maintaining asepsis. | 10 | 168 | 1 | 9.88 | 0.41 |
| Secure body wall closures. Consistently closes dead space. Good skin to skin apposition with subcuticular patterns. | 10 | 168 | 1 | 9.71 | 0.67 |
| **Professionalism/Behavior (*pass/fail*)** |  | | | | |
| Treats all animals with respect. Handles animals appropriately. | 1 or 2 | 168 | 1 | 1.00 | 0.00 |
| Arrives on time | 1 or 2 | 168 | 1 | 1.00 | 0.00 |
| Pitches in to get all the work done. | 1 or 2 | 168 | 1 | 1.00 | 0.00 |
| Positive friendly attitude | 1 or 2 | 168 | 1 | 1.00 | 0.00 |
| Acts professionally. Positive representative of Mississippi State and the Veterinary Profession. | 1 or 2 | 168 | 1 | 1.00 | 0.00 |

| **Surgical Skills** |  | **Academic Year 2015-16** | | | |
| --- | --- | --- | --- | --- | --- |
| **Question** | **Scale** | **Total Answered** | **Total Unanswered** | **Mean** | **SD** |
| Understands the basics of each procedure at the start of the rotation (i.e. has thoroughly reviewed the PowerPoints and videos). | 10 | 161 | 69 | 9.69 | 0.60 |
| Applies what is learned each day to subsequent surgeries, i.e. doesn't repeat mistakes. | 10 | 223 | 7 | 9.27 | 0.82 |
| Thinks about what he/she is doing. Pays attention to detail. Performs task appropriately. | 10 | 227 | 3 | 9.35 | 0.76 |
| Adopts efficient techniques. Shows improvement in efficiency over the course. | 10 | 221 | 9 | 9.08 | 1.00 |
| Effectively prevents hemorrhage. | 10 | 227 | 3 | 9.28 | 0.75 |
| Treats tissues gently. | 10 | 226 | 4 | 9.35 | 0.77 |
| Pays attention to maintaining asepsis. | 10 | 227 | 3 | 9.68 | 0.65 |
| Secure body wall closures. Consistently closes dead space. Good skin to skin apposition with subcuticular patterns. | 10 | 227 | 3 | 9.51 | 0.74 |
| **Professionalism/Behavior (*pass/fail)*** |  | | | | |
| Treats all animals with respect. Handles animals appropriately. | 1 or 2 | 227 | 3 | 1.00 | 0.00 |
| Arrives on time. | 1 or 2 | 227 | 3 | 1.00 | 0.00 |
| Pitches in to get all the work done. | 1 or 2 | 227 | 3 | 1.00 | 0.00 |
| Positive friendly attitude. | 1 or 2 | 226 | 4 | 1.00 | 0.00 |
| Acts professionally. Positive representative of Mississippi State and the Veterinary Profession. | 1 or 2 | 227 | 3 | 1.00 | 0.00 |

**Supplementary Table 4.** Results of Objective Structured Assessment of Fourth-year Students for Two Academic Years. For the pass/fail questions, a score of 1 = pass, 2 = fail.
